# Editorial Correspondence

**Published:** 1873-07

**Authors:** W. F. Westmoreland

**Affiliations:** Rome, Georgia


### EDITORIAL CORRESPONDENCE.

ROME, GEORGIA, July 6th, 1873.

*Dear Journal*,—I arrived in Rome last night, and to-day had the pleasure of an interview with Miss J——, the lady upon whom Dr. Robt. Battey performed the operation of "normal ovariotomy" last August. As this case has been freely discussed, and involves questions of grave interest, I have thought that my interview would not be uninteresting to the readers of the JOURNAL.

To my surprise I found Miss J—— presenting all the evidences of the most perfect health—bright eyes, rosy cheeks and an apparent vitality, which indicate just the reverse of mental and physical suffering. I was informed by the young lady and her aunt that for fifteen or sixteen years before the operation her sufferings were most intense—in fact, greater than recorded by Dr. B. in his report of the case; that she never was entirely exempt from suffering. As the young lady expressed it, she felt there was no future for her in this life, and that death would be a relief. For the past several months she has never suffered a pain. She has, however, had a flow from the womb resembling the menstrual flux, every two to four weeks, up to six weeks ago, since which time she has seen nothing. I will not stop to discuss the probable character of this discharge, as time alone must determine this physiological problem.

Suffice it to say that Miss J—— is apparently, and by her statements, in the most perfect health; that from ninety to one hundred pounds in weight, she has increased to one hundred and thirty-five pounds, presenting all the appearances of a healthy lady of thirty years of age.

I have been more impressed with the probable future of "normal ovariotomy" as practiced by Dr. Battey, since seeing and conversing with this his first case, than I could have been by pages descriptive of the great change effected in her condition by the operation. I hope the Doctor will make other efforts in the same direction, as the results in this case are sufficiently encouraging.

Truly, W. F. WESTMORELAND.

